# Improving Complex Coacervate Tissue Adhesive Performance Using Bridging Polymer Chains

**DOI:** 10.1021/acs.biomac.4c01801

**Published:** 2025-03-25

**Authors:** Ayla N. Kwant, Julien S. Es Sayed, Nawal Aledlbi, Hanna Pryshchepa, Pieter J. van der Zaag, Janette K. Burgess, Dirk-Jan Slebos, Simon D. Pouwels, Marleen Kamperman

**Affiliations:** † Polymer Science, Zernike Institute for Advanced Materials (ZIAM), University of Groningen, Groningen 9747AG, The Netherlands; ‡ Department of Pathology and Medical Biology, University of Groningen, University Medical Center Groningen, Groningen 9713GZ, The Netherlands; § Groningen Research Institute for Asthma and COPD, University of Groningen, University Medical Center Groningen, Groningen 9713GZ, The Netherlands; ∥ Department of Pulmonary Diseases, University of Groningen, University Medical Center Groningen, Groningen 9713GZ, The Netherlands; ⊥ Department of Biomedical Engineering, University of Groningen, University Medical Center Groningen, Groningen 9713GZ, The Netherlands; # Department of Gastroenterology and Hepatology, University of Groningen, University Medical Center Groningen, Groningen 9713GZ, The Netherlands; ¶ Zernike Institute for Advanced Materials (ZIAM), University of Groningen, Molecular Biophysics, Groningen 9747AG, The Netherlands; ∇ Department of Nuclear Medicine and Molecular Imaging, University of Groningen, University Medical Center Groningen, Groningen 9713GZ, The Netherlands

## Abstract

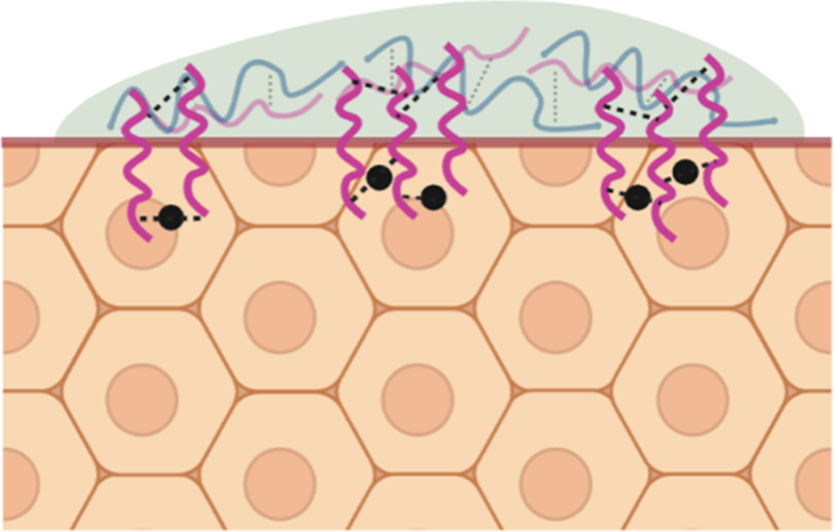

Complex coacervates have emerged as promising tissue adhesives due to their excellent wet adhesion and tunable properties. However, maintaining stable adhesion on soft, dynamic tissues remains challenging. In this study, the use of a bridging polymer was investigated to enhance the adhesive properties of a complex coacervate adhesive (CCA) composed of poly(allylamine hydrochloride) (pAH) and polysulfopropyl methacrylate (pSPMA). The CCA undergoes solidification as a result of a change in salt concentration, forming a robust adhesive under physiological conditions. Pretreatment with pAH, but not pSPMA, significantly improved adhesion energy on both model hydrogels and biological tissues by forming a polymer-rich bridging layer at the interface. The beneficial effect was driven by accumulation of pAH in superficial layers of both the CCA and the substrates. This enabled the CCA to withstand higher deformation before adhesive failure. These findings underscore the potential of bridging polymers to improve CCAs and other tissue adhesives for biomedical applications.

## Introduction

Tissue adhesives are useful materials for wound closure and bleeding control after surgical procedures, offering an alternative to invasive methods like sutures, staples, and wires. Most surgical sealants consist of cyanoacrylates, which are often associated with cytotoxicity and are incompatible with internal usage or usage on wet mucosal tissues.^1^ On the other hand, natural adhesives such as fibrin- and albumin-based sealants are associated with poor bonding strength, fast degradation, allergic reactions, and disease transmission.^2^

Another class of tissue adhesives which has recently emerged is the class of complex coacervates.^3^ Complex coacervates are formed through liquid–liquid phase separation (LLPS) that results from electrostatic association between oppositely charged polyelectrolytes in aqueous solution.^4^ Upon LLPS, two phases are formed: the supernatant, which is depleted of polymers, and the complex coacervate phase, which is rich in polymers. Complex coacervates are good adhesives under wet conditions, as they exhibit a low interfacial tension with water, enabling them to spontaneously spread on a submerged surface without dissolving in water.^5^ Furthermore, they have tunable mechanical properties ranging from free-flowing liquid to elastic solid, making them suitable as adhesives on a range of tissues and materials, including bone, cartilage, and skin tissue, as well as synthetic implants.^5^ In vitro and in vivo testing has shown that complex coacervates show good biocompatibility and aid in tissue repair.^6,7^

One of the unique characteristics of complex coacervates is that their mechanical properties and their processability can readily be tuned by a set of environmental conditions. The ionic strength of the environment has the most striking effect.^8,9^ At high ionic strength, polymer–salt interactions are the most predominant, while polymer–polymer interactions are weak. At low ionic strength, with less polymer–salt interactions, association between the polymers in the coacervate is promoted. This enables the production of complex coacervates with low viscosity at high salt concentrations, which can solidify after delivery to the desired site by undergoing a so-called “salt switch”.^10^ This in situ solidification and hardening has been shown to be efficient to turn complex coacervates into load-bearing tissue adhesives.^11,12^

Maintaining a stable bond between adhesive and tissue is critical, especially when working with soft and dynamic tissues. Human tissues such as muscles, heart, and lungs are constantly in motion, undergoing different types of deformation. For the adhesive to remain functional, it should be able to maintain contact with the tissue surface, despite different deformations. Recently, it has been shown that the interfacial bond between an adhesive hydrogel and a tissue substrate can by strengthened by using bridging polymers.^13,14^ Polycations containing primary amines, like chitosan and polyallylamine (pAH), were used to create a bridging layer between hydrogels and tissues, through self-association by electrostatic interactions, hydrogen bonding, covalent bonds, and physical interpenetration.^1,15^

Using this “molecular stitching” approach, a tough hydrogel-based adhesive was designed that was able to adhere strongly to several wet tissue substrates, yielding large improvement in adhesion.^16^ It has not been investigated yet, whether a similar strategy can also be utilized to improve the adhesion of complex coacervate adhesives (CCAs). The beneficial properties of complex coacervates, combined with bridging polymers, would provide us with a unique wet adhesive that is able to maintain a stable bonding to biological tissues. We hypothesized that using bridging polymers can be a useful strategy to improve the adhesion strength of complex coacervates. In this study, for the first time, the benefits of using bridging polymers to provide better load-bearing adhesive performance of a CCA were investigated.

## Experimental Section

### Materials

Sulfopropyl methacrylate potassium salt (SPMA), 4-cyano-4-(phenylcarbonothioylthio)pentanoic acid (CTP), 4,4′-azobis(4-cyanovaleric acid) (ACVA), acrylamide (AAm), *N*,*N′*-methylenebis(acrylamide) (MBA), ammonium persulfate (APS), *N*,*N*,*N*′,*N*′-tetramethylethylenediamine (TEMED), rhodamine B isothiocyanate (RITC), 4% formaldehyde, benzyl benzoate (BB), and benzyl alcohol (BA) were purchased from Sigma-Aldrich (Darmstadt, Germany). Poly(allylamine hydrochloride) (pAH, molecular weight: 50 – 100 kg/mol) and 4′,6-diamidino-2-phenylindole (DAPI) were purchased from Thermo Fisher (MA, United States). Methacryloxyethyl thiocarbamoyl rhodamine B (MTRB) was purchased from Polyscience Inc. (PA, United States). Phosphate buffer saline (PBS) was purchased from Gibco (MA, United States). All chemicals were used as received without further purification. Deionized water was produced by reverse osmosis (conductivity <10 μS cm^–1^ ≈ 10^–4^ M NaCl). Tissues were obtained from a local butcher shop (Nazar, Groningen, The Netherlands).

## Methods

### pSPMA Synthesis

pSPMA was obtained by reversible addition–fragmentation chain transfer (RAFT) polymerization of SPMA in water. SPMA (2.000 g, 8.120 mmol), CTP (0.015 g, 0.054 mmol), ACVA (0.0032 g, 0.0116 mmol), and 5 mL of deionized water were successively added in a 10 mL glass round-bottom flask. To obtain rhodamine B-labeled pSPMA (rho-pSPMA), MTRB (0.0054 g, 0.0008 mmol) was added. The targeted degree of polymerization (DP) was set to 151. The solution was degassed with N_2_ for 5 min at room temperature (RT) to remove dissolved oxygen. Then, the reaction was allowed to run for 16 h at 70 °C. The final monomer conversion was determined to be 100%, as confirmed by ^1^H NMR analysis before and after freeze-drying (Figure S1). After full conversion, the pSPMA chains with an average DP of 151 units were calculated to have a molecular weight (*M*_n_) of *M*_n_ = DP × *M*_SPMA_ + *M*_CTP_ = 151 × 246.32 + 279.38 g/mol ≈ 37,500 g/mol. Finally, the mixture was freeze-dried to yield pSPMA as a white fluffy powder (red for rho-pSPMA).

### Fluorescent Labeling of pAH

pAH (2.00 g, 21.51 mmol of repeat units) was dissolved in 40 mL of deionized water in a 100 mL glass round-bottom flask. The pH was adjusted to 9 using a 1 M NaOH solution. RITC (0.011 g, 0.021 mmol) was then directly dissolved in the solution. The reaction was allowed to run at RT for 24 h. The rhodamine B-labeled pAH (rho-pAH) was further purified by dialysis against deionized water for 3 days, renewing the dialysis bath with fresh deionized water twice per day. The completion of the purification process was determined when the surrounding dialysis bath did not exhibit the presence of RITC (determined by UV–visible spectroscopy). Finally, the rho-pAH was obtained as dry powder by freeze-drying (yield = 65%). Successful labeling of both pSPMA and pAH was measured by UV–visible spectroscopy, in which the characteristic absorption peak of the rhodamine B around 550 nm can be observed (Figure S2).

### pAAm Hydrogel Preparation

pAAM was synthesized using the following protocol, which was based on a previous protocol reported by our group.^17^ AAm (2.280 g, 32 mmol), MBA (0.0048 g, 0.032 mmol), APS (0.0044 g, 0.020 mmol), and 8 mL of deionized water were successively added in a 20 mL vial. The hydrogel precursor solution was degassed with N_2_ for 5 min to remove the dissolved dioxygen. Next, 60 μL (0.4 mmol) of TEMED was added under stirring to trigger the pAAm polymerization. After homogenization, the solution was injected into a mold consisting of two rigid plexiglas walls and a 3 mm silicone spacer and allowed to polymerize overnight at RT. The obtained hydrogel was removed from the mold and hydrated in 50 mL PBS for 16 h to reach a final thickness of around 5 mm.

### Complex Coacervate Preparation

The pAH/pSPMA CCA was prepared with an equal concentration of positively and negatively charged units of 0.5 M and a final NaCl concentration of 2 M. First, stock solutions of pAH and pSPMA (2.7 M of charged units), of which the pH was adjusted to 7 using a 1 M NaOH solution, were prepared separately. pSPMA is a strong polyelectrolyte, so all SPMA repeat units are charged and the degree of ionization is α- = 1. pAH is a weak polyelectrolyte; so, its degree of ionization at pH 7 was calculated to be α+ = 98.4%, following the formula below (p*K*_a_ = 8.8).^18^ Taking the degree of ionization and molecular weight into account, the appropriate mass of both polyelectrolytes was determined to reach an equal amount of charged units.
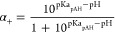


The pSPMA stock solution was then mixed with deionized water and a 5 M NaCl solution. Subsequently, the pAH stock solution was added, and the mixture was immediately vortexed. The mixture was then mixed further on a tube rotator for 15 min, after which it was centrifuged at 4500 *g* for 15 min at RT. After centrifuging, the dense sedimented complex coacervate phase was separated from the supernatant.

### Complex Coacervate Characterization

The change in total mass upon salt switch was determined by weighing 150 μL of unswitched CCA. This was subsequently submerged in excess PBS and allowed to solidify for 24 h, after which the total mass of the CCA was determined again.

Thermogravimetric analysis (TGA) was performed to assess the water, polymer, and salt content of the CCA before and after salt switch. The sample before salt switch was measured immediately after preparation, and the salt-switched sample was allowed to solidify for 24 h in excess PBS (>1000:1 PBS to sample ratio). TGA was performed on a TA Discovery series 5500 under continuous airflow. Samples were heated from 20 to 700 °C at a heating rate of 20 °C min^–1^ without prior weight stabilization. The results are expressed in weight percentage (wt %) of the initial sample weight.

Viscoelastic measurements and probe tack adhesion measurements on the CCA before and after salt switch were performed on an Anton Paar MCR302e rheometer. The samples after salt switch were allowed to solidify for 24 h in PBS before performing the measurement. All viscoelastic measurements were performed using a 25 mm diameter cone–plate geometry with a 1° angle (CP25-1). Frequency sweeps were performed at a fixed strain of 1% from 100 to 0.10 rad s^–1^. The strain of 1% lies well within the linear viscoelastic regime for both conditions (before and after salt switch).

Probe tack adhesion measurements were performed using a 10 mm diameter sandblasted plate–plate geometry (PP10-S). Unswitched samples were used as prepared, loaded onto the stainless-steel bottom plate of the rheometer, and the geometry was lowered to an initial sample thickness of 150 μm. After a waiting time of 30 s, the probe was retracted with a speed of 100 μm s^–1^, recording the normal force as a function of displacement. To study the adhesive properties after switching, samples were loaded similarly and the probe was lowered to 150 μm. PBS was then added such that the sample, in contact with the probe, was submerged and allowed to solidify for 15 min. The probe was then retracted at the same speed as for unswitched samples. The work of adhesion (*W*_adh_) was calculated by dividing the area under the force–displacement curve by the contact area of adhesion.

### Cytocompatibility Testing

Cytocompatibility of the CCA was determined using a commercially available cancer cell line (A549). Testing was conducted using the material prepared at a NaCl concentration of 2.1M. Cells were seeded in 24-well plates and cultured at 37 °C, 5% CO2 until confluent. Cells were then exposed to (1) 25 μL of the material directly or (2) an extract of the material, prepared according to ISO 10993-5 guidelines for biological evaluation of medical devices.^19^ After 24 h of exposure, cell viability and activity was assessed using an MTS assay (Promega, WI, United States). As positive control, 10% Triton X-100 in 70% ethanol was used to achieve 100% cell death. After sufficient color development, absorbance was measured at 490 nm (Epoch 2 Microplate reader, BioTek, VT, United States). Cell viability was calculated by subtracting the absorption values for the positive control and expressing viability as % of the untreated control group.

### Tensile Adhesion Measurements

Adhesion measurements were performed on an Instron Universal Testing System (68SC-1). pAAm hydrogels and muscular tissues were cut to 15 × 15 mm squares with a thickness of 5 mm. The pieces were attached to T-shaped brass rods that were cut to 15 × 15 mm dimensions, using Superglue (Bison, The Netherlands). For pAH or pSPMA, 50 μL of solution (10 mg mL^–1^ in deionized water, pH adjusted to 7 using 1 M NaOH) was added to the surface of the tissue or hydrogel. After 5 min, substrates were loaded onto the tensile tester. Next, 75 μL of CCA was added onto the bottom hydrogel/tissue piece, after which the pieces were compressed together immediately (0.1 N for tissue, 0.5 N for hydrogel). After 15 min of solidification of the CCA, tensile measurements were performed with a constant pulling speed of 5 mm min^–1^. The *W*_adh_ was calculated by dividing the area under the force–displacement curve by the contact area of adhesion.

### Confocal Scanning Laser Microscopy on pAAm Hydrogels

The diffusion of rho-pAH and rho-pSPMA (10 mg mL^–1^, pH 7) inside pAAm hydrogels swollen with PBS was followed by confocal scanning laser microscopy (CLSM) using a Stellaris 8 (Leica, Germany). A 543 nm laser (laser intensity = 6%, digital gain = 100%) was used for excitation of rhodamine B and the emitted light was retrieved in a wavelength-window between 550 and 650 nm. A 10× objective lens (numerical aperture, NA = 0.75, dry immersion) was used for measurements. In a typical measurement, the hydrogel (15 × 15 mm square) was placed on top of a microscopy cover slide, and a 10 μL drop of either rho-pAH or rho-pSPMA solution was added on the surface of the hydrogel. Z-stack measurements were then immediately performed (scan speed = 1000 Hz, resolution = 512 × 512 pixels, scan area = 77.5 × 77.5 μm, *z*-traveling distance = 500 μm, *z*-sectioning = 10 μm, total acquisition time per measurement = 22 s) from the top surface of the hydrogel to *z* = −500 μm inside the hydrogel. Measurements were performed at *t* = 0.5, 1, 2, 3, 5, 7, 9, 11, 13, and 15 min. For each measurement or diffusion time, the average fluorescence intensity as a function of the depth in the hydrogel was measured using Fiji software (option “Plot *Z*-axis profile”) and reported as a value normalized by the maximum fluorescence intensity of at each diffusion time.^20^

### Dynamic Light Scattering

The average scattering intensity (mean count rate, kHz) of 1 mg mL^–1^ pAH and pSPMA solutions in PBS was measured by dynamic light scattering (DLS) at a detection angle of 90°, using a Zetasizer Ultra (Malvern Panalytical, United Kingdom) equipped with a HeNe laser (λ = 632.8 nm). All analyses were performed with the software supplied by the manufacturer.

### 2D Fluorescent Microscopy

To study the diffusion of bridging polymers into the CCA or biological tissues, 10 mg mL^–1^ solutions of rho-pAH or rho-pSPMA were used. For this purpose, skeletal muscle tissue was used. 30 μL of the polymer solution was added on top of a piece of tissue (dimensions 10 × 5 mm, with 5 mm thickness), or a piece of solidified CCA of similar dimensions. After 5 min, samples were carefully washed with PBS to remove any unattached polymers. Next, the samples were fixed in paraformaldehyde (PFA) (Sigma-Aldrich) overnight. Fixed samples were embedded in paraffin and sliced into 4 μm sections using a microtome. The glass slides with sections were deparaffinized using two 10 min incubations in xylene (Klinipath BV, The Netherlands), and xylene was removed using 100% ethanol before mounting the slides using Aquatex (Merck). Fluorescent microscopy was performed with the EVOS Cell Imaging System (Thermo Fisher, MA, United States).

### 3D Fluorescent Microscopy with Cleared Tissue

Tissues were sectioned into slices of uniform thickness (ranging from 3 to 5 mm). Prior to the tissue clearing process, 30 μL of labeled primer (of rho-pAH or rho-pSPMA, 10 mg mL^–1^) was applied directly onto the surface of each tissue sample and left to penetrate for 10 min. Subsequently, the tissues were rinsed three times with PBS for 10 min to ensure thorough washing. Samples were then incubated in 4% formaldehyde for 24 h to fix and stabilize the tissue structure, minimizing potential degradation during subsequent clearing steps. After fixation, tissues were rinsed in PBS twice for 15 min.

To stain the nuclei, the tissue sections were immersed in a DAPI solution (0.5:1000 μL in PBS) and incubated overnight on a shaker (VWR Standard 1000 Standard Orbital Shaker) to promote uniform staining. Next, samples were washed in PBS overnight to remove excess DAPI.

To dehydrate the tissues, each sample was sequentially immersed in ethanol solutions of increasing concentrations: 25%, 50%, 75%, and finally 100%. Each dehydration step lasted 2 h. Subsequently, samples were placed in a 1:1 mixture of ethanol and BABB (BABB is a 2:1 mixture of benzyl alcohol and benzyl benzoate) for 2 h with continuous shaking to ensure complete immersion and to facilitate the transition to a more transparent state.^21^

Finally, the refractive index within the samples was matched to ensure uniformity between different components. This was achieved by incubating the samples in BABB for an additional 2 h to achieve maximum transparency. All incubation steps were conducted on a shaker in 5 mL Eppendorf tubes filled with the relevant solution to maintain consistent exposure.

3D images were acquired by CLSM using a Leica Stellaris 8 SP8, with similar settings as imaging in the pAAm hydrogel. The scan speed was set to 600 Hz. A 405 nm laser was used for the excitation of DAPI, and the emitted light was retrieved in a wavelength window between 426 and 485 nm. 3D images were processed using Leica LAS X 3D Visualization.

### Statistics

All statistical analyses were performed using GraphPad Prism 10.3.1. Pairwise comparisons were performed by Mann–Whitney U tests. ns = not significant, * = *p* ≤ 0.05, ** = *p* ≤ 0.01. Probe tack and tensile adhesion measurements were performed with *n* = 5–6 for each condition. Values are reported in the text as mean ± SD.

## Results and Discussion

### Complex Coacervate Adhesive

**Figure 1:**
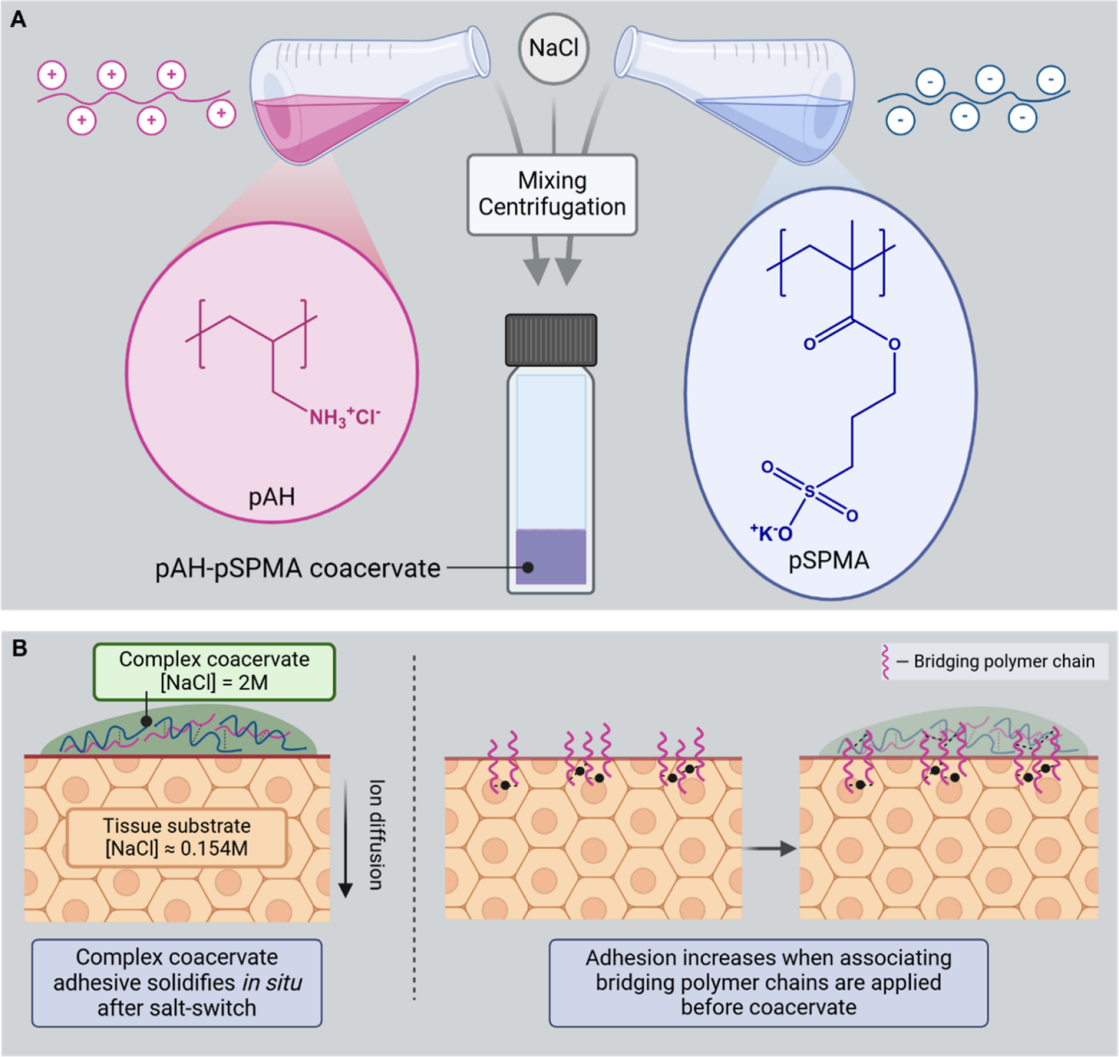
(A) Schematic of the production of the pAH/pSPMA CCA. (B) Schematic of the solidification of the CCA as a result of the salt switch and the improvement of adhesion using bridging polymer chains. Figure was created with BioRender.com.

The CCA used in this study consisted of pAH as a polycation and oppositely charged polysulfopropyl methacrylate (pSPMA) as a polyanion (Figure 1A). pAH and pSPMA were chosen for the CCA, as they have a backbone exclusively composed of carbon–carbon covalent bonds to avoid any unwanted material degradation, which is an important feature when considered for use as a long-term in vivo adhesive. The bottom phase resulting from LLPS is the CCA; the supernatant phase on top was not used. The CCA was liquid-prepared at a NaCl concentration of 2 M, which was easily injectable through an 18G needle (Supplementary Video 1). The CCA underwent a salt switch to solidify and become an adhesive material (Figure 1B). As potential bridging polymers, both polyelectrolytes were used in a single-polymer aqueous solution (Figure 1B).

**Figure 2:**
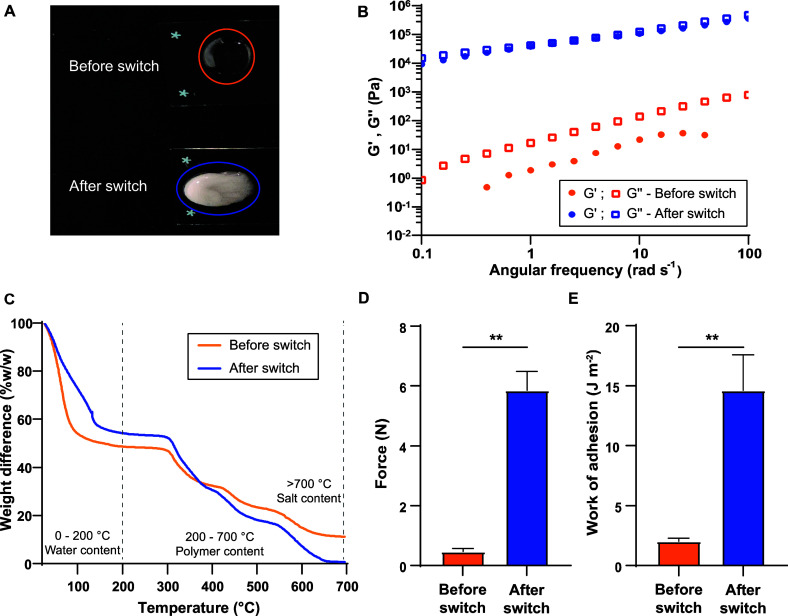
Complex coacervate characterization before and after salt switch. (A) Photo of a droplet of CCA on a glass microscope slide. (B) Frequency sweep performed on CCA. (C) Thermogravimetric analysis (TGA) performed on CCA. (D) Maximum force (*F*_max_) measured by tack adhesion measurements on the CCA. (E) Work of adhesion (*W*_adh_) measured by tack adhesion measurements on the CCA. Data shown as mean ± SD with *n* = 5 per condition. Statistical differences were tested using a Mann–Whitney *U* test, **p ≤ 0.01.

The solidification of the CCA was investigated by immersing it in PBS. Immediately upon immersion, the adhesive turned into an opaque and white solid (Figure 2A). This transition of visual appearance is commonly reported for complex coacervates that undergo a salt switch.^22^ This solidification is a result of the release of salt from the CCA, leading to a sudden strengthening of the electrostatic interactions between the polyelectrolytes. Due to the local high polymer concentration, the release rate of water that is originally hydrating the polyelectrolyte chains in the coacervate is drastically hindered. This in turns leads to the formation of microscopic water pockets, sometimes named “pores”, dispersed within the polymer-rich hardened matrix.^22^ The strongly scattering white color arises from the coexistence of these two microscopic phases with dissimilar refractive index inside the CCA.

To confirm the liquid-to-solid transition of the CCA, rheological measurements were performed (Figure 2B). Before the salt switch, the CCA showed the rheological characteristics of a low viscosity free-flowing viscoelastic liquid with its loss modulus (G″) overpassing its storage modulus (G′) over the whole range of frequencies investigated. After the initial solidification upon submerging the CCA in PBS, both G′ and G″ values increased by 5 and 4 orders of magnitude, respectively. Additionally, G′ and G″ values were almost similar over the entire range of frequencies investigated, indicating the formation of a critical gel with characteristic dissipative properties that are often sought for in pressure-sensitive adhesives.^12,23^

The total mass of the complex coacervate decreased by 27.8% upon salt switching. This mass loss is due to the release of salt and water, which is usually observed for complex coacervate systems.^11,24^ The change in composition after the CCA underwent a salt switch was confirmed by thermogravimetric analysis (TGA) (Figure 2C). The first decrease in weight (0–200 °C) corresponds to the evaporation of the water present in the material, which is 51.3% for the unswitched sample and 45.7% for the switched adhesive. It is commonly reported that when coacervate systems lose salt, in this case due to the salt switch, there is a lower water content too.^25^ The next decrease in weight (200–700 °C) is attributed to the degradation of organic matter, corresponding to the polymer content of the material. The polymer content was 37.5% before salt switch and 53.4% after salt switch. The mass that is left after the material has been heated to 700 °C is the NaCl. The NaCl content before the salt switch is 11.2%, which closely corresponds to the 2 M NaCl concentration that was used for preparing the CCA. After solidification, the NaCl content decreased to 0.9%, confirming the ion release. In PBS, the NaCl concentration is also 0.9%, indicating the salt release from the CCA is complete.

Next, the beneficial effect of CCA solidification on the adhesive performance was investigated by probe tack adhesion measurements on model stainless steel surfaces of a rheometer. The unswitched materials showed poor adhesive performance, with a maximum force (*F*_max_) of 0.46 ± 0.10 N and a work of adhesion (*W*_adh_) of 2.03 ± 0.25 J m ^–2^ (Figures S3 and 2D,E). After the material was solidified, the adhesive performance significantly increased, with a *F*_max_ of 5.86 ± 0.63 N and *W*_adh_ of 14.61 ± 2.98 J m ^–2^. Measured *W*_adh_ values are in the same range of previously reported complex coacervate-based adhesives, after undergoing solidification in a physiological environment.^11,12^ The salt switch that makes this material an adhesive is an important advantage over conventionally used cyanoacrylates, which rely on polymerization reactions that lead to inflammation and damage.^26^ It is important to note that while the salt switch can be an advantage over cyanoacrylates in terms of cytotoxicity, the adhesive values reported in this study are lower than those of cyanoacrylates. Cytocompatibility of our CCA was assessed by MTS assay (Figure S4). According to ISO 10993-5, the material can be considered noncytotoxic, as the relative cell viability upon direct exposure is higher than 70%. Exposure to the extract, however, was slightly cytotoxic (67.4% viability relative to control).

Taken together, a CCA prepared from pAH and pSPMA is minimally cytotoxic and undergoes a salt switch upon being submerged in PBS, inducing an increase in viscoelastic moduli and adhesion.

### Adhesion and Diffusion of Bridging Polymers in a pAAm Hydrogel Model Substrate

**Figure 3:**
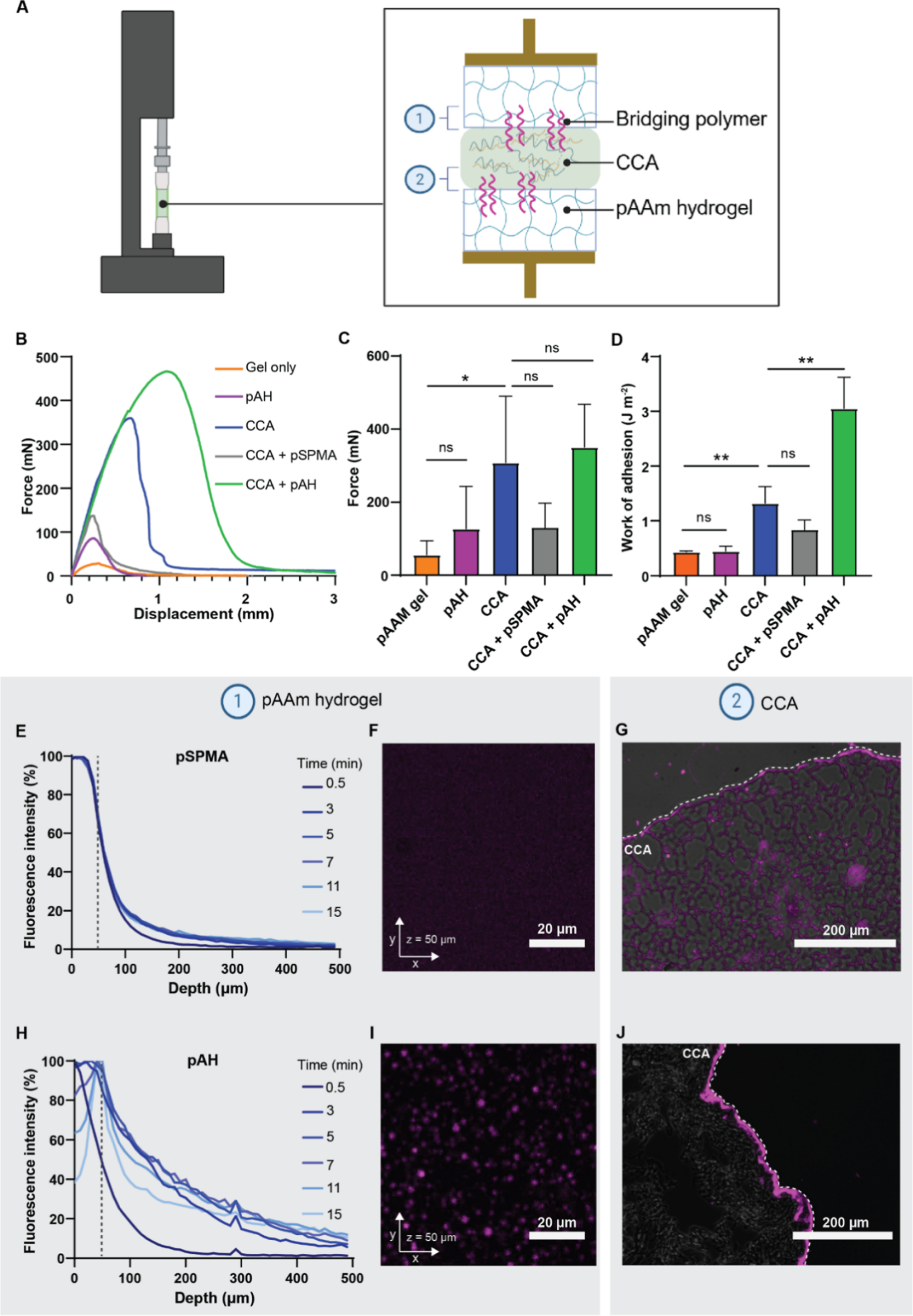
Adhesion and diffusion of bridging polymers in a PBS swollen pAAm hydrogel model substrate (1) and CCA (2). (A) Schematic representation of the experimental setup of adhesion measurements, performed on a tensile tester. Note: The scale of this image is not representative of the real experiment. (B) Representative force–displacement curves of tensile adhesion measurements, of pAAm hydrogel substrates alone or treated with CCA and/or bridging polymers. (C) Maximum force (*F*_max_) measured by tensile adhesion measurements. (D) Work of adhesion (*W*_adh_) measured by tensile adhesion measurements. Data shown as mean ± SD with a *n* = 6 per condition. Statistical differences were tested using a Mann–Whitney test, *p ≤ 0.05, **p ≤ 0.01. Diffusion profile in pAAm hydrogels at different time points of (E) fluorescent pSPMA and (H) fluorescent pAH solutions. Images obtained at 50 μm depth for hydrogels treated with (F) fluorescent pSPMA and (I) fluorescent pAH solutions at the 15 min time point. Images of microsections of solidified CCA, showing the diffusion profile after 15 min diffusion time of (G) fluorescent pSPMA and (J) fluorescent pAH in the CCA, the white dashed line indicates the surface of the CCA.

Next, the effect of bridging polymers on the adhesive properties of the pAH/pSPMA CCA was investigated. The bridging polymers tested were single-polymer solutions of pAH and pSPMA, added to the substrate before application of the CCA. Adhesion of the CCA was measured using a tensile tester (Figure 3A). pAAm hydrogels swollen in PBS were used as the adhesive substrate, as they form a uniform and wet surface without any biological variation of tissues. After compression, the CCA formed a uniform layer with a thickness of about 1–1.5 mm.

Two untreated pAAm gels, without addition of CCA or bridging polymer, showed negligible adhesion (*F*_max_ = 55.6 ± 38.8 mN, *W*_adh_ = 0.44 ± 0.02 J m ^–2^) (Figure 3B–D). Pretreatment of both pAAm hydrogel surfaces with an aqueous solution of pAH (10 mg mL^–1^, pH 7) resulted in a slightly increased *F*_max_, yet the overall adhesive performance was not significantly increased. In agreement with the previous observations, the CCA alone significantly increased both the *F*_max_ and *W*_adh_ (*F*_max_ = 307.7 ± 182.7 mN and *W*_adh_ = 1.33 ± 0.30 J m^–2^). Whitening and hardening of the CCA were observed, evidencing the successful salt switch of the CCA in contact with the pAAm hydrogel substrate (Figure S5). The pretreatment of the pAAm gel with pSPMA (10 mg mL^–1^, pH 7) as the bridging polymer combined with the CCA appeared to slightly decrease *F*_max_ or *W*_adh_, although not significant. This could be because of the hindered contact between substrate and adhesive. However, when the pAAm gel was pretreated with pAH as the bridging polymer before application of the CCA, the *W*_adh_ increased significantly to 3.05 ± 0.57 J m ^–2^. The increase in *W*_adh_ is obtained because pretreatment with pAH allows the CCA to withstand a higher displacement before the adhesive detaches from the hydrogel (Figure 3B). Regardless of whether the CCA was used alone or in combination with pAH pretreatment, ultimately, an adhesive debonding occurred at the top surface (Figure S6). The force (350.5 ± 117.3 mN) required to displace or detach the material did not increase, as this is mainly controlled by the bulk stiffness of the CCA.^27−29^ This beneficial effect requires a combination of the pretreatment with pAH solution and the CCA, as the pAH solution alone does not alter the adhesive properties of the CCA on pAAm gels.

The mechanism by which pAH enhances the adhesive performance of the CCA on the model pAAm hydrogel substrate immersed in PBS was further investigated by CLSM, fluorescent optical microscopy, and DLS. For the microscopy observations, pAH and pSPMA were chemically labeled with rhodamine B to assess their ability to diffuse in both the pAAm hydrogel substrate and the CCA (Figure 3E–J). The evolution of relative fluorescence intensity, which is directly proportional to the local concentration of labeled polymer, was measured as a function of penetration in the PBS swollen pAAm hydrogel for a diffusion time up to 15 min (Figure 3E,H). Limited diffusion within the hydrogel was observed for both polymers. Nevertheless, some key differences can be highlighted. In the case of pSPMA, no evolution of the fluorescence intensity in the hydrogel after 3 min of diffusion time was observed. In the case of pAH, although diffusion is limited, the maximum fluorescence intensity appeared to gradually move over time from the surface, where the drop was deposited, to the layers just beneath the surface of the hydrogel. At 7 min of diffusion time, a clear fluorescence peak was observed at a depth of 50 μm. At this depth, additional images were taken after 15 min of diffusion time, to visualize the bridging polymer deposition (Figure 3F,I). For pSPMA, no specific pattern was observed with the fluorescent signal homogeneously dispersed throughout the *X*–*Y* plane, and for pAH, fluorescent aggregates were observed at a depth of 50 μm in the hydrogel. These results indicate that the diffusion of both polymers is primarily limited by topological effects due to the tight mesh size of the pAAm network. However, in the case of pAH, aggregates are observed just below the surface of the hydrogel.

DLS measurements were performed to investigate the aggregation behavior of pAH and pSPMA. Whereas pAH showed strong aggregation when dispersed in PBS, pSPMA did not show any sign of aggregation (Figures S7 and S8). The aggregates were observed to eventually sediment, which is characteristically observed for larger particles, of which the size cannot reliably be determined by DLS. Nevertheless, an indicative size of 2–5 μm can be estimated from the CLSM images of pAH-rich aggregates in pAAm hydrogel in Figure 3I. This pAH chain aggregation phenomenon in PBS has already been reported in the literature, in which specific ionic and hydrogen bonding interactions between primary amines and phosphate ions were proven to be advantageous when used as nanocarriers for drug delivery.^30,31^ These interactions likely lead to physical entanglement of the pAH-phosphate aggregates into the substrate, leading to increased load transmission.

Diffusion of pAH and pSPMA chains into the CCA was also investigated. To do so, fluorescent microscopy images were taken of microsections of the solidified CCA that was treated with labeled polymers beforehand (Figure 3G,J). The CCA treated with pSPMA showed a weak signal throughout the whole CCA, indicating that some of the pSPMA remained bound to the CCA after washing with PBS, without being located specifically at the application surface (Figure 3G). On the other hand, pAH appeared to form a bridging layer at the surface of the CCA, with a thickness of approximately 10 μm (Figure 3J). Similar to the pAAm hydrogel, the pAH chains appear to accumulate in the subsurface layers of the CCA. Thorough washing did not disrupt this layer, indicating that the accumulated pAH chains are entangled into the CCA network. As the CCA was solidified in PBS, it is likely that the bridging layer formation is due to the aggregation of pAH with phosphate, as is the case in the pAAm hydrogels. This layer formation was not observed for pSPMA.

Taken altogether, these results highlight the beneficial effects of using pAH as a bridging polymer between a model hydrogel substrate and the CCA. While pAH forms a bridging layer on both sides of the adhesive joint, this is not observed for pSPMA. This bridging is likely due to the ionic association of pAH with phosphate ions. The formation of this bridge between the two sides leads to the formation of polymer-dense load-bearing domains, or “molecular stitches”, which enable the CCA to withstand higher levels of extension before detachment occurs. This can explain why pAH, but not pSPMA pretreatment, enhanced CCA adhesion.

This mechanism is reminiscent of the so-called topological adhesion approach that has been reported earlier. In these studies, self-association of chitosan, poly(4-aminostyrene), or cellulose inside hydrogels was also advantageously used to increase the adhesion between two hydrogels.^13,16^ As an example, in the case of chitosan, a similar limited diffusion profile inside the hydrogels was observed above pH 7 where intermolecular hydrogen bonds between chitosan chains are predominant.

### Adhesion and Diffusion of Bridging Polymers on Biological Tissue

**Figure 4:**
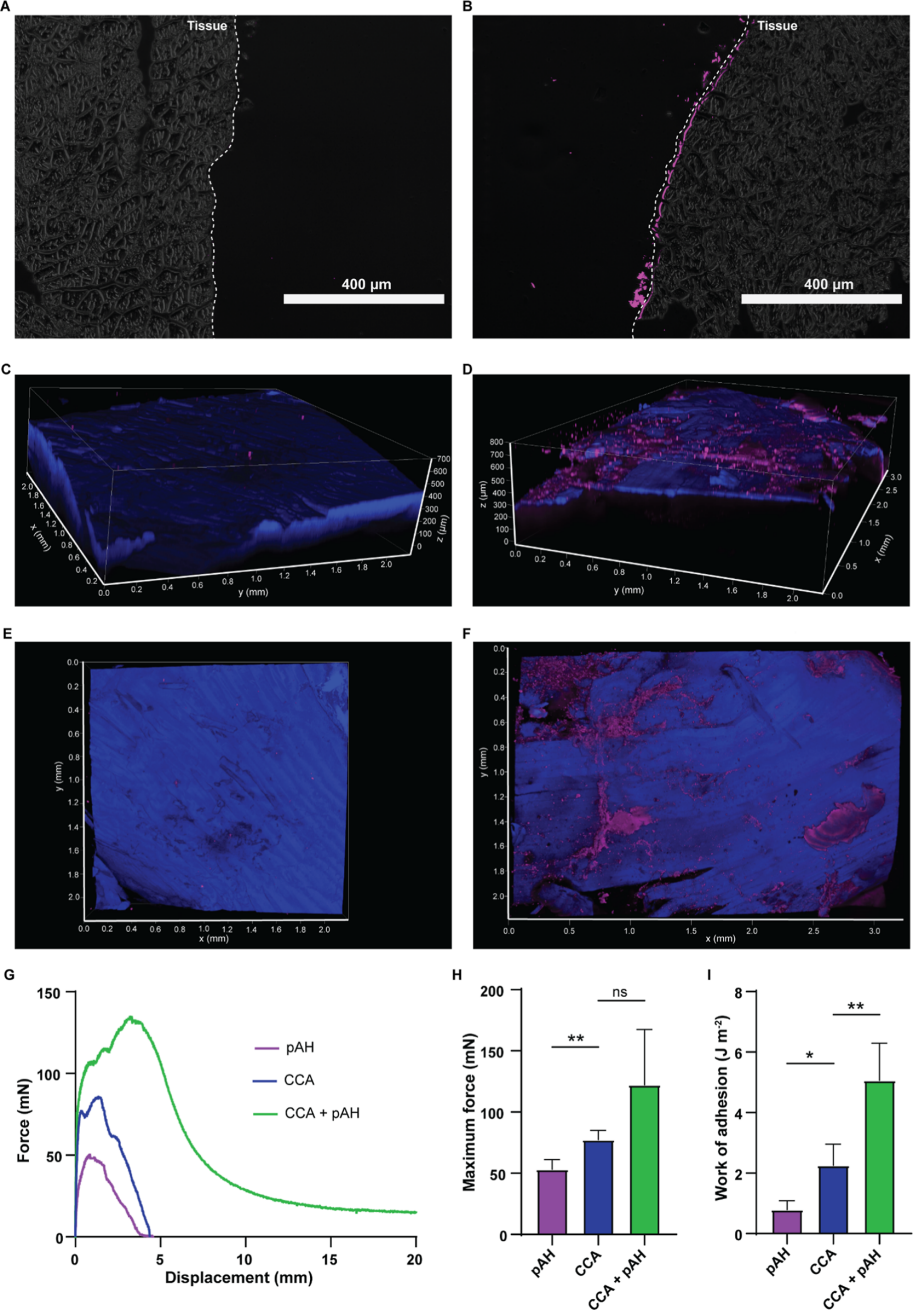
Diffusion and adhesion of bridging polymers on biological tissue. 2D images of microsections of skeletal muscular tissue with (A) fluorescent pSPMA and (B) fluorescent pAH deposited on the surface, the white dashed line indicates the application surface of the fluorescent polymers on the tissue surface. 3D images of skeletal muscular tissue with (C + E) fluorescent pSPMA and (D + F) fluorescent pAH deposited on the surface, obtained using tissue clearing. Nuclei were stained with DAPI in blue. (G) Representative force–displacement curves of tensile adhesion measurements. (C) Maximum force (*F*_max_) measured by tensile adhesion measurements. (D) Work of adhesion (*W*_adh_) measured by tensile adhesion measurements. Data shown as mean ± SD with *n* = 5 per condition. Statistical differences were tested using a Mann–Whitney *U* test, *p ≤ 0.05, **p ≤ 0.01.

Next, the beneficial effects of combining the CCA with pAH as the bridging polymer were investigated using biological tissues, increasing the translational potential of our data. Fluorescently labeled pAH and pSPMA were deposited onto skeletal muscular tissue to observe their diffusion profile. Thorough washing was performed to remove any unbound polymer. After fixation, embedding and slicing, no signal was detected in the samples treated with labeled pSPMA solution (Figure 4A). This indicates that no pSPMA chains aggregated in or bound to the tissue, and all the polymers were washed away before the sectioning process. On the other hand, labeled pAH formed a layer at the application surface at a depth of 5–10 μm (Figure 4B). This suggests that pAH, like in the pAAm hydrogel and CCA, aggregates in biological tissue to form a bridging layer.

To get a more complete view of the tissue, the same tissues were also investigated in their entirety, without slicing. A huge challenge when looking at whole tissues is that samples thicker than 4–6 μm are generally almost impossible to visualize using CLSM.^32^ By tissue clearing, the penetration depth of microscopy was improved by making the tissues translucent. In this study, a mixture of benzyl alcohol and benzyl benzoate (BABB) was utilized as an optical tissue clearing agent, which works by removing the lipids from the tissue.^33^ Labeled pAH and pSPMA were added to the muscular tissue surface, and after washing away any unbound polymer, the tissues were successfully cleared (made transparent) (Figure S9). The 3D CLSM data (Figure 4C–F, Supplementary Videos 2 and 3) indicated that pAH remained localized on the tissue surface after thorough washing of the sample (Figure 4D). Precise penetration depth measurements were hindered by surface heterogeneity and the presence of cracks, allowing the polymer solution to enter these areas. When looking at the top view (Figure 4F), pAH appears to be located at the tissue surface in localized regions, suggesting heterogeneity in affinity across the tissue surface. In the case of pSPMA, there appears to be no affinity to the tissue at all. There was barely any labeled polymer detected at the tissue surface (Figure 4C,E), indicating that little polymer remained bound to the tissue upon washing. These data are consistent with the 2D images of the same tissues, confirming that pAH stays bound to the tissue surface with limited diffusion, while pSPMA does not form this bridging layer.

The bridging layer of pAH in skeletal muscular tissue, observed by 2D and 3D imaging, is likely also an effect of phosphate-induced aggregation, for example, with the phosphate present in the cell membranes. Phosphate is essential in the human body, where it is an important constituent of various cellular components like phospholipid membranes, nucleic acids, and intracellular signaling proteins.^34^ Furthermore, electrostatic interactions may also have an additional effect on the formation of this polymer bridging. The positively charged amine groups of pAH (degree of ionization at pH 7 = 98.4%) can interact with negatively charged cell membranes and extracellular matrix components, which can contribute to the observed effect.^35,36^

To confirm whether the bridging of pAH increased adhesion on skeletal muscular tissue, tensile adhesion testing was performed (Figure 4G–I). As is the case in pAAm hydrogel substrates, pretreatment with pAH had a beneficial effect on adhesion in skeletal muscular tissue substrates. The recorded force–displacement curves of the samples pretreated with pAH before CCA application were much wider compared to those of the samples with only CCA (Figure 4G). In fact, the adhesive did not detach from the tissue substrate at the end of the measurement (displacement = 20 mm), when the tissue was pretreated with pAH. This resulted in a significant increase in *W*_adh_ (from 2.26 ± 0.70 to 5.06 ± 1.23 J m ^–2^) (Figure 4H), while there was no significant difference in *F*_max_ (Figure 4I). The increased adhesion was only observed when pAH solution and CCA were combined, as pAH without CCA showed low average adhesion values (*F*_max_ = 53.2 ± 8.0 mN, *W*_adh_ = 0.79 ± 0.30 J m ^–2^).

It must be noted that the *F*_max_ and *W*_adh_ recorded on muscular tissue were lower than the values recorded on pAAm hydrogel substrates, especially when the CCA was applied. This could be the result of an incomplete solidification, due to a lack of moisture in the tissue sample. A lack of moisture may impair the diffusion of salt out of the CCA and into the tissue, leading to the sample not fully switching. In an in vivo situation, where circulation constantly keeps the water content of tissues stable, this effect could be overcome. Furthermore, the adhesion energy measured is strongly affected by the toughness, stiffness, and strength of the substrate, as well as the surface homogeneity, making different substrates difficult to quantitatively compare to each other.^1^ This also explains the difference in values compared to other works that report the use of bridging polymer chains in tissue adhesives.^13,16^ The hydrogels used in these studies have a high bulk toughness, automatically relating to higher recorded *W*_adh_. Our CCA is not in the same range of adhesive force of conventional cyanoacrylates. Therefore, the CCA would be most useful in applications where a high peak force is not necessary or recommended, such as sealing of organ leaks or skin wounds, where mostly a high extensibility without failure is essential.

Despite the lower recorded forces, pAH was also able to increase the *W*_adh_ on tissue substrates, without an increase in *F*_max_. Our data indicate that pAH, but not pSPMA, can aggregate in muscular tissue as well, forming load-bearing domains. This helps the CCA to withstand a higher displacement before the adhesive detaches from the tissue surface. This observed improved ability to maintain contact with the adhesive substrate is beneficial for a tissue adhesive. Biological tissues are dynamic, and it is therefore important that an adhesive can withstand deformation. This would prevent adhesive failure and associated applications like severe bleeding, infections, and inadequate healing.^37^ The enhanced *W*_adh_ may also improve the durability of the bond, reducing the need for replication of the adhesive.

The application of bridging polymers may be extrapolated to other complex coacervates as well. There is a large range of CCAs that are being investigated for various medical applications, including bleeding control, wound healing, and internal organ sealing.^38−40^ Not only pAH but also chitosan, poly(4-aminostyrene), and cellulose could form a bridging layer that strongly increases *W*_adh_, although these have not yet been investigated in combination with CCAs.^13,16^ This is the first study to report the combination of a CCA with bridging polymer chains, and while the results are promising, the absolute values recorded are still relatively low. It would be interesting to further study the combination of CCAs and bridging polymers, for example, to see whether the composition of the CCA influences the effectiveness of the bridging polymer, and what the influences are of molecular weight and concentration of the bridging polymer. Further optimizing this system could optimize and improve adhesive values, which can benefit the development of CCAs, with the prospective to use them in a clinical setting.

## Conclusions

In the present study, the effect of using bridging polymers on the adhesive performance of a pAH/pSPMA CCA was investigated. The CCA is a liquid that undergoes a salt switch, solidifying upon contact with physiological conditions. This environmental trigger turns the liquid CCA into an adhesive material. When studying the effect of bridging polymer chains on CCA adhesion, it was found that pAH, but not pSPMA, formed aggregates in both sides of the adhesive joint, likely due to ionic interactions. The formation of this “bridge” between the hydrogel or tissue substrate and CCA surface significantly improved *W*_adh_, indicating the formation of load-bearing domains that facilitates the CCA to withstand a higher displacement before adhesive failure.

Tissue adhesive failure can lead to major complications, such as severe bleeding, which is why the formation of a stable bond between adhesive and tissue is crucial, even when deformation occurs. Maintaining adhesion without exerting disproportionate forces to the surrounding tissue can prevent pain or damage, improving patient comfort. The use of bridging polymer chains will aid a tissue adhesive to maintain contact with the substrate, resisting detachment under dynamic conditions without excessive peak forces. This is a promising strategy to improve currently available tissue adhesives, as well as for other CCAs which are currently being investigated for a large range of applications.

## Supporting Information Available

Additional images, NMR spectra, and data on visible spectrometry, tack adhesion, cytocompatibility, and DLS (PDF)
Injection video and 3D images of skeletal muscular tissue with fluorescent pAH and pSPMA deposited on the surface, using tissue clearing (ZIP)
