# Supplementary material for: Improving Complex Coacervate Tissue Adhesive Performance Using Bridging Polymer Chains
Source: Biomacromolecules. 2025 Mar 25;26(4):2433–43. doi: 10.1021/acs.biomac.4c01801 (PMC12004509; doi:10.1021/acs.biomac.4c01801)
Supplement: Supplementary file 1 — bm4c01801_si_001.pdf [file bm4c01801_si_001.pdf]

Supporting information for *"Improving Complex Coacervate Tissue Adhesive Performance Using Bridging Polymer Chains"*

Ayla N. Kwant,[a,b,c,d] *Julien S. Es Sayed,*[a,e] *Nawal Aledlbi,*[f,g] *Hanna Pryshchepa,*[a] *Pieter J. van der Zaag,*[g,h] *Janette K. Burgess,*[b,c] *Dirk-Jan Slebos,*[c,d] *Simon D. Pouwels,*[b,c,d] *Marleen Kamperman*[a] *

[a] University of Groningen, Polymer Science, Zernike Institute for Advanced Materials (ZIAM), Groningen, The Netherlands

[b] University of Groningen, University Medical Center Groningen, Department of Pathology and Medical Biology, Groningen, The Netherlands

[c] University of Groningen, University Medical Center Groningen, Groningen Research Institute for Asthma and COPD, Groningen, The Netherlands

[d] University of Groningen, University Medical Center Groningen, Department of Pulmonary Diseases, Groningen, The Netherlands

[e] University of Groningen, University Medical Center Groningen, Department of Biomedical Engineering, Groningen, The Netherlands

[f] University of Groningen, University Medical Center Groningen, Department of Gastroenterology and Hepatology, Groningen, The Netherlands

[g] University of Groningen, Molecular Biophysics, Zernike Institute for Advanced Materials (ZIAM), Groningen, The Netherlands

[h] University of Groningen, University Medical Center Groningen, Department of Nuclear Medicine and Molecular Imaging, Groningen, The Netherlands

* Corresponding author - marleen.kamperman@rug.nl

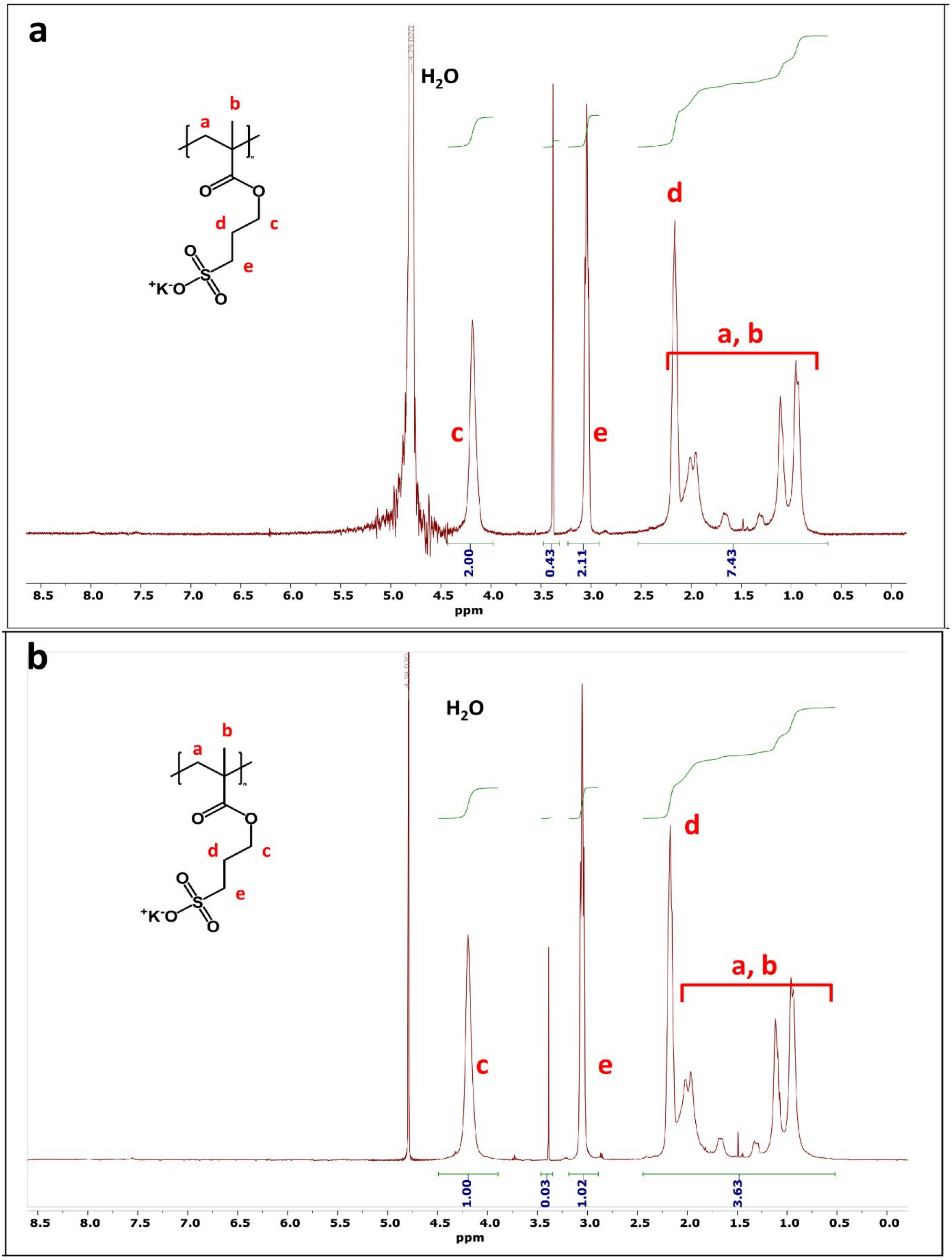

Figure S1. ^{1}H NMR in D_2O of polysulfopropyl methacrylate (pSPMA) after 16 hours of polymerization **a)** before and **b)** after freeze drying. The absence of peaks at 6 -7 ppm indicates complete conversion of the monomer.

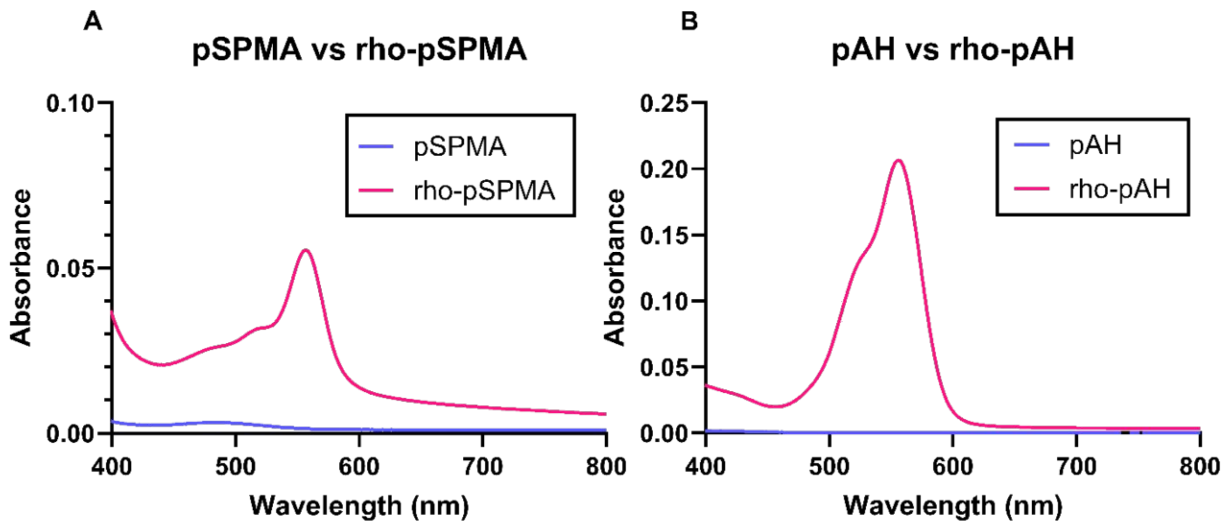

Figure S2. Absorbance curves measured by visible spectroscopy of 1 mg mL^{-1} aqueous solutions of **a)** unlabeled (pSPMA) and rhodamine B labeled polysulfopropyl methacrylate (rho-pSPMA) and **b)** unlabeled (pAH) and rhodamine B labeled polyallylamine hydrochloride (rho-pAH). Both show the characteristic absorption peak of rhodamine B around 550 nm.

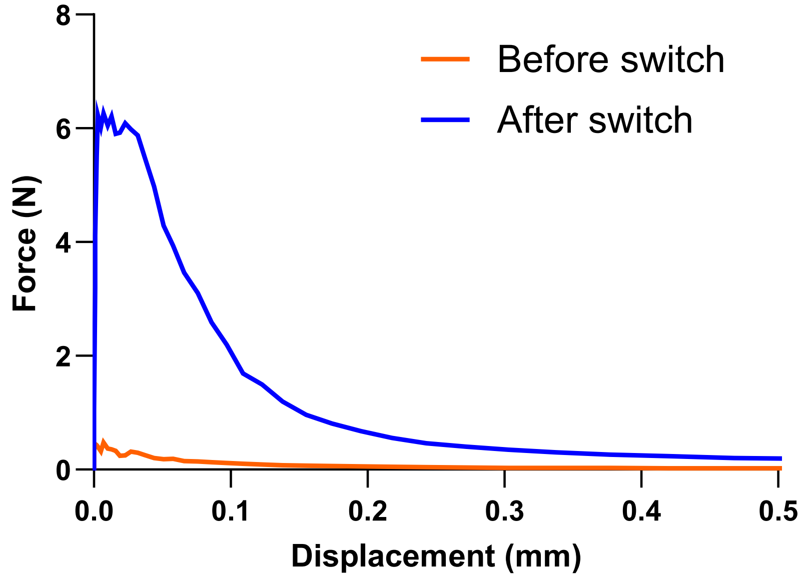

Figure S3. Representative force-displacement curves, measured by probe tack adhesion measurements on the complex coacervate adhesive before (orange) and after (blue) salt switch.

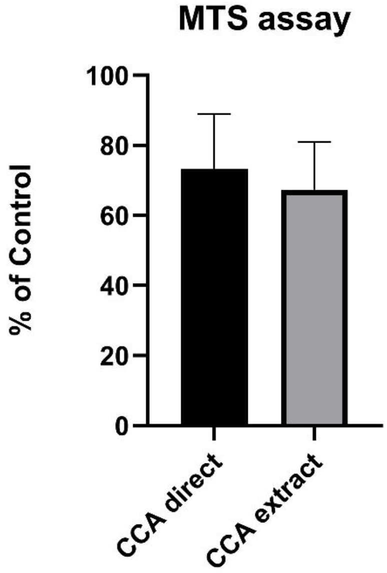

Figure S4. MTS assay of A549 cells exposed to the complex coacervate adhesive (CCA) directly or by extraction. Viability was expressed as % of untreated control. Data shown as mean ± SD with n=3 per condition.

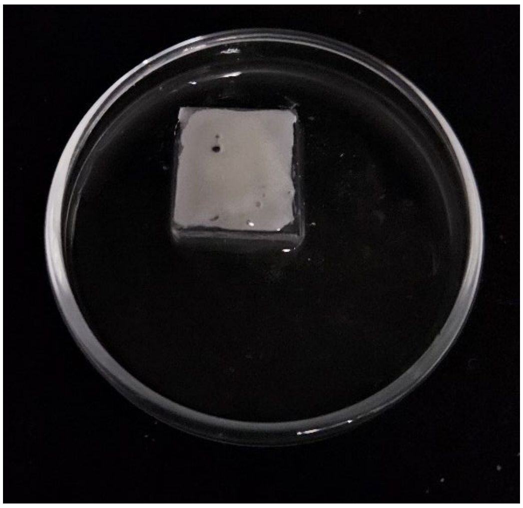

Figure S5. Photo of opaque solidified complex coacervate adhesive (CCA), as a result of the successful salt switch between the CCA and the polyacrylamide hydrogel substrate of lower ionic strength.

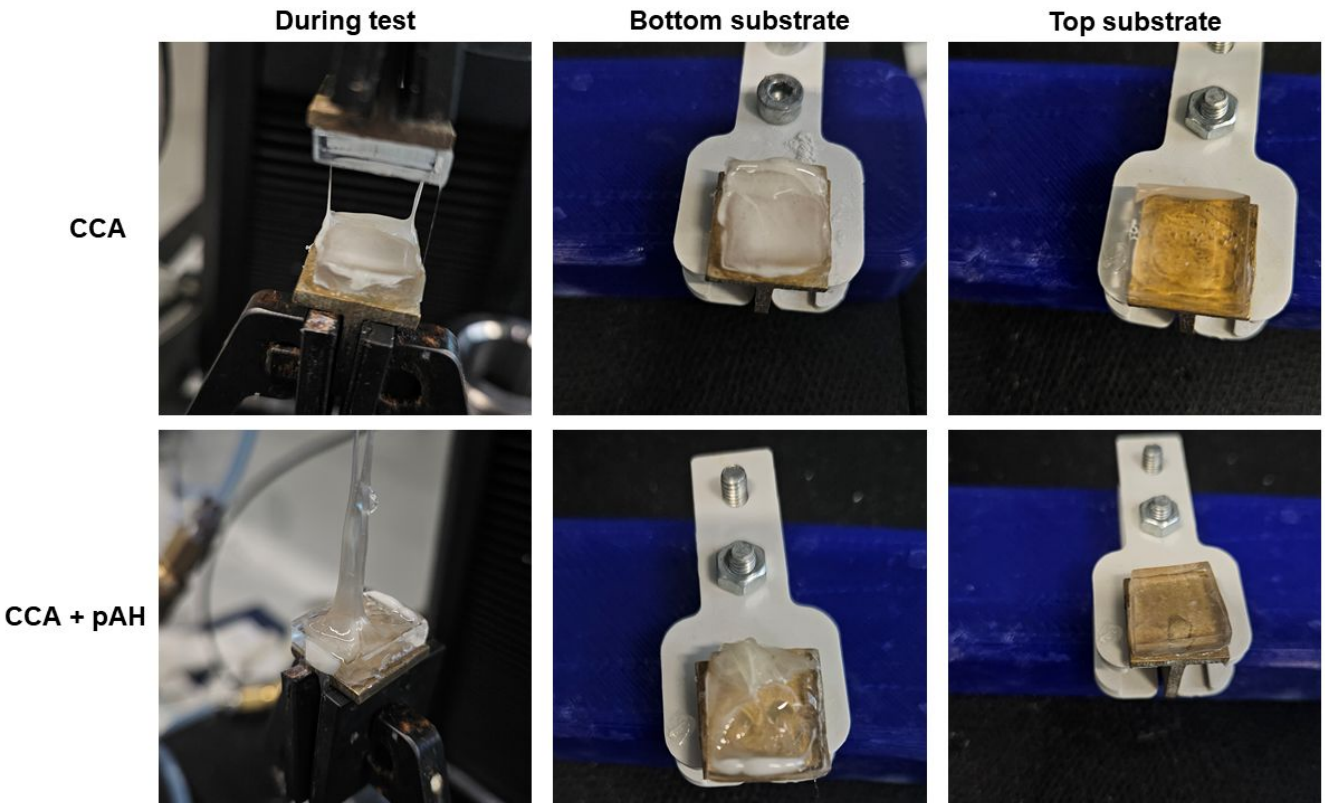

Figure S6. Images demonstrating the mode of adhesive failure of the complex coacervate adhesive (CCA) with or without pretreatment with polyallylamine hydrochloride (pAH) on pAAM hydrogel substrates. Images were taken during the tensile test, as well as after adhesive failure.

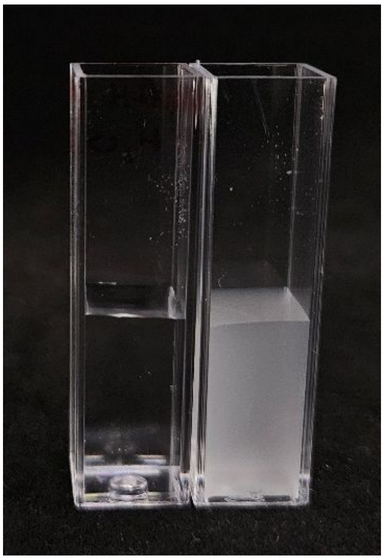

Figure S7. Photo of 1 mg mL^{-1} polyallylamine hydrochloride (pAH) and polysulfopropyl methacrylate (pSPMA) solutions in phosphate buffered saline (PBS). Aggregation can be observed for pAH but not for pSPMA.

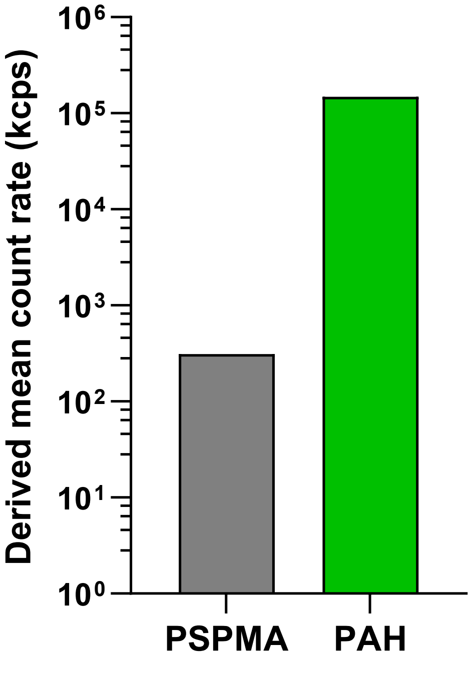

Figure S8. Representative intensities of scattered light by 1 mg mL^{-1} polysulfopropyl methacrylate (pSPMA) (grey) and polyallylamine hydrochloride (pAH) (green) solutions in phosphate buffered saline (PBS). Intensity of scattered light is expressed in kilo-counts per second (kcps), measured by dynamic light scattering (DLS).

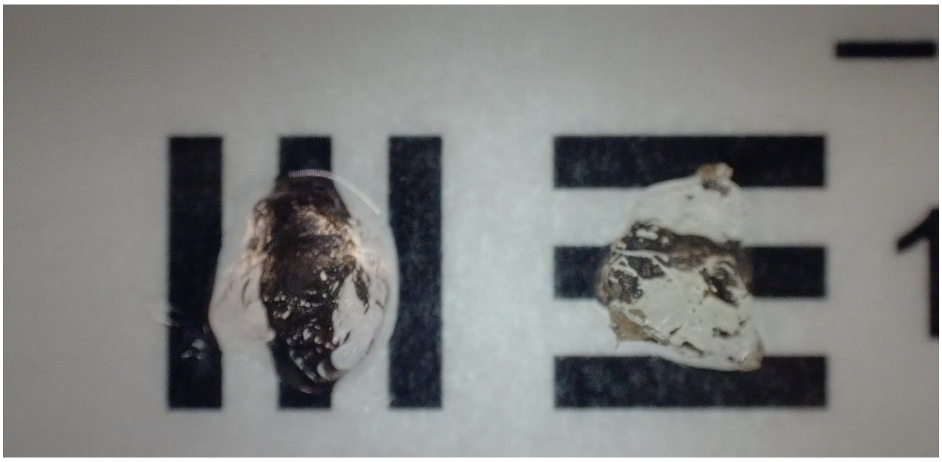

Figure S9. Photo of the muscular tissue samples that were successfully made transparent using benzyl alcohol and benzyl benzoate (BABB). The left sample was treated with polyallylamine hydrochloride (pAH), while the right sample was treated with polysulfopropyl methacrylate (pSPMA).
